# Dementia risk prediction in the general population: external validation of a prediction model in the population-based LifeLines Cohort Study

**DOI:** 10.1016/j.jarlif.2025.100028

**Published:** 2025-09-26

**Authors:** Ingeborg Frentz, Sofia Marcolini, Silvan Licher, Peter Paul De Deyn, Mohammad Arfan Ikram

**Affiliations:** a Department of Epidemiology, Erasmus MC, Rotterdam, the Netherlands; b Department of Neurology, UMCG, Groningen, the Netherlands; c Department of General Practice, Erasmus MC, Rotterdam, the Netherlands; d Laboratory of Neurochemistry and Behavior, Experimental Neurobiology Unit, University of Antwerp, 2610 Antwerp, Belgium

**Keywords:** Dementia, Alzheimer, Prediction, Prognosis, Primary care

## Abstract

**Background:**

Models for dementia prediction in primary care are necessary to identify individuals at risk for developing dementia, but their implementation in clinical practice is partly limited due to lack of external validation or use of high-cost variables. We externally validated the predictive performance of a simple yet promising dementia risk prediction model.

**Methods:**

We assessed discriminative ability with a *c*-statistic with 95 % confidence interval, using age, history of stroke, subjective memory complaints and need for assistance with a relatively complex task as predictors. This was done on 10,007 individuals that participated in the Lifelines-cohort study. Assessment of dementia in the Lifelines Cohort Study is self-reported in the follow-up questionnaires.

**Results:**

Mean follow-up at LifeLines timepoint 1b was 1.5 years, mean follow-up at LifeLines timepoint 2a was 3.3 years and mean follow-up at LifeLines timepoint 3a was 9.1 years. Overall, 36 participants self-reported dementia development. Discriminative ability of the model overall dementia development yielded a *c*-statistic of 0.62 [95 % CI=0.48–0.70], and performed slightly better at follow-up 2a 0.67 [95 % CI=0.57–0.78]. However, calibration of the model in this external validation cohort was poor, with systematic overestimation of the predicted risk.

**Conclusion:**

In this study the basic dementia risk prediction model overestimated the risk of dementia, but had reasonable discriminative ability in the Lifelines cohort. Within this validation cohort the potential of the model is underestimated due to low incidence of reported dementia. Further validation is required to determine the true value of the model. Studies assessing its implementation feasibility in primary care should also be conducted.

## 1 Introduction

Worldwide the number of people with dementia is rising, and substantial increases in overall prevalence are predicted due to prolonged life expectancy [1,2]. Care and support of patients with dementia has a high burden on families, health-care systems and society [3]. While no definite cure for dementia is available yet, many of the manifestations associated with dementia are now known to be manageable and disease course could be modifiable with good dementia care [4]. Additionally, there is growing evidence that lifestyle interventions can contribute to delaying dementia onset [1,3]. Earlier diagnosis of dementia and the identification of persons at risk of developing dementia is important to implement lifestyle changes that could reduce the risk of developing dementia or slow disease progression [5]. The focus on early detection is supported by the findings that some dementia cases can be prevented to a certain extent if the currently known modifiable risk factors are eliminated [6,7].

Through prediction modelling with known risk factors for dementia, an individual’s probability of developing all-cause dementia over a specified time can be estimated. Such models can be used to determine individuals at high-risk of developing disease, inform personalized forecasting of disease development, and could improve selection of individuals for future clinical trials and preventive interventions [[8], [9], [10]]. Predictive accuracy of models with high-cost variables, such as MRI parameters, a wide range of cognitive assessments and blood-based biomarker screenings, is higher than those with only routinely collected variables [11,12]. However, the costs restrain the use of such models especially in a primary care setting. There have been some models developed using primary-care information [[12], [13], [14]]. But, the lack of the models’ external validation poses a limit to their application in the general population [15]. Three models that are slightly more extensive than only primary-care information have been developed and also externally validated, and have been implemented into clinical trials and practice [[16], [17], [18]]. Further refinement of the application of the models would be beneficial.

For a prediction model to be applied in a primary care setting, the model would mainly use already available or very easily obtained information [11]. The basic prediction model developed within the Rotterdam Study includes only four variables to calculate the risk of developing dementia. The included predictors were: age, history of stroke, presence of subjective memory decline, and need for assistance with finances or medication [19]. Because this model includes only four predictors it could be easily implemented to calculate risk of developing dementia.

For every model external validation is essential to assess the generalizability, to determine whether the model can be applied to a wider population than the one from which it was developed [1,10]. Therefore, this study aimed to evaluate the performance of the basic prediction model of all-cause dementia that can be used in primary care settings, developed in the Rotterdam Study [19]. In this study we validated this model in an independent population-based cohort: the LifeLines Cohort Study. While external validation is a necessary prerequisite for implementation of a model, assessing its implementation feasibility was not analyzed in this study.

## 2 Material and methods

### 2.1 Variables of the prediction model

The significant predictors of the basic primary care dementia prediction model developed within the Rotterdam Study include only four variables to calculate the risk of developing dementia. Similar to most prediction models, it includes age as a predictor, the Rotterdam study model then included presence of subjective memory decline as a predictor, the prevalence of this predictor in the general population increased predictive performance of the model. They additionally used history of stroke as predictor instead of individual cardiovascular risk factors, as vascular risk factors are important in the development of dementia. However, the role of separate risk factors can diminish with increasing age, reducing specific discriminative ability for prediction of dementia development. The final predictor of this model is need for assistance with finances or medication [19]. Variables used in the current study were matched as closely as possible to the variables of the Rotterdam Study model. For age, history of stroke and presence of subjective memory complaints an exact match was available; for the need for assistance variable we used the question: “Do you think it is difficult to follow health recommendations regarding for example diet, physical activity, lifestyle or medication intake?”. In the Rotterdam study the participants were asked about medication use and financial management by trained interviewers with a questionnaire about instrumental activities of daily living (IADL). As the question for the “need for assistance” variable differed from the original model variable we defined a score based on the answers given in the questionnaire. Based on the answers, the weight of the score was calculated in four parts from 0 to 1 (based on the four answers options to the question), with 0 meaning no difficulty with the task and 1 meaning they found the task very difficult.

### 2.2 Independent validation cohort

The LifeLines Cohort Study, a large population-based cohort study in the northern provinces of the Netherlands, was started in 2006 to investigate environmental, genetic, behavioural, physical and psychological factors that may contribute to health and disease. Details on the Lifelines study have been previously published [20,21]. In brief, between 2006 and 2013, 10 % (around 167 000) of the northern population of The Netherlands aged 0 to 93 years was enrolled using a three-generation recruitment design. Data was collected through questionnaires (all ages) and measurements (age 8 years and older) on physical and mental health, lifestyle and the exposome. Participants completed questionnaires every 1.5 years and had follow-up visits every 5 years [20,21]. Assessment of dementia is included in the questionnaire as self-reported incidence, participants are presented with the question if dementia has developed since the previous questionnaire.

For the present study, we included 10,162 participants who had data on subjective memory complaints. We excluded 73 participants who had no information on “history of stroke”, 8 that had no information on “need for assistance with finances or medication” and 3 that had dementia at baseline. Finally, 71 people younger than 65 were excluded. The remaining sample consisted of 10,007 participants. Assessment of dementia in the Lifelines Cohort Study is self-reported and part of the follow-up questionnaire. In this questionnaire participants are asked if health problems have started since the last time they filled in the Lifelines questionnaire, dementia is one of the health problems listed that participants can fill in. Within Lifelines data collection is separated in questionnaire-only and on-site visits which include a physical examination and questionnaires, these follow-up examinations are classified in several timepoints: baseline assessment (timepoint 1a), questionnaire follow-up (timepoints 1b and 1c), second physical examination (timepoint 2a), questionnaire follow-up (timepoint 2b) and third physical examination (timepoint 3a). For this study there were three follow-up times included, timepoints 1b, 2a and 3a. Mean follow-up time at timepoint 1b was 1.5 years, mean follow-up at timepoint 2a was 3.3 years and mean follow-up at timepoint 3a was 9.1 years. Currently, data collection for follow-up time 3a is ongoing, thus a smaller sample was available to determine model performance at 3a.

### 2.3 Statistical analysis

Participant characteristics were analysed using descriptive statistics. To calculate the risk of dementia we applied the original regression equation of the model. In the original model by Licher *et al*. hazard ratios for the predictors of the model were presented [19]. Using these hazard ratios (age = 1.09, history of stroke = 1.82, subjective memory decline = 1.31 and need for assistance = 1.46) we were able to calculate a linear predictor which subsequently was used to calculate predicted risk of dementia development.

Formula to calculate the linear predictor is as follows:

Linearpredictor=0.089680×(Age−71.2)+0.601269×HistoryofStroke+0.267845×SubjectiveMemoryComplaints+0.380281×NeedforAssistance

The formula to determine the calculated risk is as follows: Calculated Risk ( %) = (1 – exp (−0.0614 × exp(Linear Predictor))) × 100 %.

Discrimination of the model was calculated with a *c*-statistic with 95 % confidence interval, we determined the fit of the model on 3 follow-up times and for overall dementia development. We produced calibration plots to visualise the relationship between the predicted probability and observed proportion of developing dementia. All statistical analyses were performed in R version 4.2.0 (R Foundation for Statistical Computing, Vienna, Austria).

## 3 Results

Table 1 shows the descriptive characteristics of the study population. The baseline characteristics of the Rotterdam Study and Lifelines cohort sample were largely similar. The mean age was 69.7 years (SD 4.4), and 5210 (52.1 %) participants were women. During follow-up, 253 participants had a history of clinical stroke and 36 developed dementia. A small part of the population reported having memory complaints (4.1 %). Of the study population 7.9 % reported having some difficulty performing a relatively complex task, while 1.0 % reported having a lot of difficulty.

**Table 1:** Population characteristics.

Study population (*N* = 10,007)	
**Age (years)**	69.7 (± 4.4)
**Female sex**	5210 (52.1 %)
**Symptomatic stroke**	253 (2.5 %)
Memory complaints	
**No**	4543 (45.4 %)
**Sometimes**	5054 (50.5 %)
**Yes**	410 (4.1 %)
**Difficulty performing relatively complex task**	
**No difficulty**	8069 (80.6 %)
**Difficult, but I can manage**	1049 (10.5 %)
**Difficult, sometimes I can manage other times not**	788 (7.9 %)
**Very difficult**	101 (1.0 %)
**Developed dementia during follow-up**	36 (0.4 %)
**Developed dementia by follow-up 1b**	<10
**Developed dementia by follow-up 2a**	21 (0.2 %)
**Developed dementia by follow-up 3a**	<10

Data are presented as frequency ( %) for categorical values, and mean ± SD for continuous variables unless indicated otherwise.

### 3.1 Discrimination and calibration

**Fig. 1:**
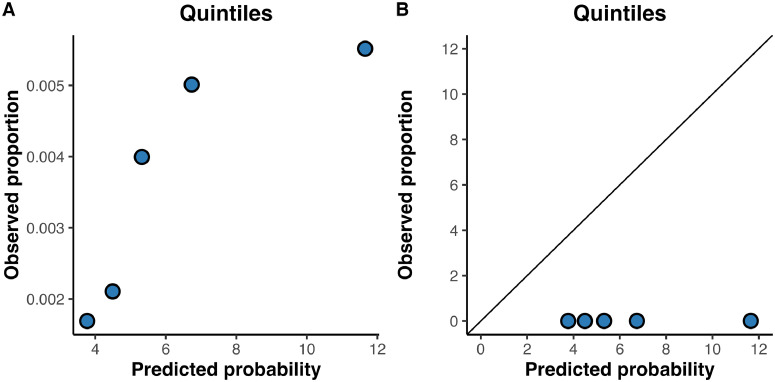
Calibration plot for the prediction model in the Lifelines validation sample.

**Legend: A.** Calibration plot of the calibrated model to predict risk of dementia in the Lifelines cohort. An intercept of 0 and a slope of 1 (the diagonal line in the right plot) represent ideal calibration. **B.** The plot on the left shows the observed proportion of dementia which is too small to be seen in the plot on the right, the plot on the right shows the fit to the ideal calibration line.

Table 2 shows the *c*-statistics for the model with confidence intervals, Fig. 1 shows the calibration plot. Calculated risk of developing dementia ranged from 3.5 % to 55.4 %, with a mean risk of 6.3 %. The model showed an overestimation of the predicted risk compared to the observed risk; there was low agreement between observed and predicted risk (Fig. 1). Overall discriminative ability yielded a *c*-statistic of 0.62 [95 % CI=0.48–0.70]. The discriminative accuracy of the model was best at follow-up time 2a, on average 3.3 years after baseline, with a *c*-statistic of 0.67 [95 % CI=0.57–0.78]. Discriminative ability at follow-up 3a was a bit lower with a *c*-statistic of 0.65 [95 % CI=0.39–0.79].

**Table 2:** Discriminative ability of the model.

Outcome	N/n	c-statistic	95 % CI
**Develop dementia overall**	10,007 / 36	0.62	0.48 – 0.70
**Develop by 1b**	10,007 / <10	0.58	0.45 – 0.80
**Develop by 2a**	10,007 / 21	0.67	0.57 – 0.78
**Develop by 3a**	1861 / <10	0.65	0.39 – 0.79

*N* = number at risk; *n* = number of events. Mean follow-up time at 1b was 1.5 years, mean follow-up at 2a was 3.3 years and mean follow-up at 3a was 9.1 years.

## 4 Discussion

This study externally validated a model for primary care dementia prediction, namely the Rotterdam dementia risk prediction model, using data from the LifeLines cohort study, to support its application in primary care and clinical trials. The discriminative ability of the model resulted to be fair with a *c*-statistic of 0.67 [95 % CI=0.57–0.78], where the discriminative accuracy of the model in the development data of the Rotterdam Study was 0.78 [95 % CI=0.75–0.81] [19]. However, calibration of the model in this external validation cohort was poor, with systematic overestimation of the predicted risk. The low calibration found in our results is possibly due to extremely low incidence of dementia reported in the study population. This might be because the follow-up time was too short to identify dementia cases, considering that data recruitment for the last follow-up time included is still ongoing. Another possible explanation for the low incidence of dementia is that in the Lifelines cohort participants may not participate in the follow-up examinations after developing dementia, potentially causing them to be lost to follow-up instead of being registered as a dementia case. Despite the fact that dementia was not the main research outcome in the Lifelines study, the discriminative accuracy of the model was fair. Overall, considering the readily available information used in this model, and the rather fair discriminative ability observed in the Lifelines Cohort, it seems that prediction of dementia development is possible using the Rotterdam Study prediction model.

Some previously developed prediction models have been implemented in clinical trials or practice. The most thoroughly studied of these models is the Cardiovascular Risk Factors, Aging and Incidence of Dementia (CAIDE) score which was converted to an app to provide guidance for individuals on risk modification [17,22]. Another model that was developed for use in public health settings without clinical assessment is the Australian National University Alzheimer’s Disease Risk Index (ANU-ADRI) [18]. While these models performed well, it was found that the CAIDE did not extrapolate well to low-and-middle income countries, and the ANU-ADRI would benefit from further refinement [22]. Three other, more basic, models have been developed specifically for use in primary care settings [[12], [13], [14]]. Of these three models only one performed well in the validation cohort [11]. While these models provide valuable tools of risk prediction, application of the primary care models is limited due to the lack of validation [11]. Although there is sufficient validation to apply the CAIDE and ANU-ADRI in practice [22], these models are still quite extensive with 8 and 12 variables included, respectively [11]. It would therefore be beneficial to have a low-cost model, with only a few variables, that can classify individuals into risk categories, so when preventive strategies for dementia have been developed it is possible to target the population at high risk of developing dementia [23]. Furthermore, accurate identification of people with higher risk of developing dementia is important because those people may be more likely to benefit from disease-modifying treatments if the intervention can be started before significant brain damage has occurred [24]. As well as disease-modifying treatments being more beneficial if they can be applied in earlier stages of disease, delaying dementia onset would greatly reduce the burden on society and increase the number of people reaching the end of life without ever developing dementia [4]. Possibly, prevention of dementia development could be better than a cure and underlies the interest in modifiable risk factors and disease prediction [4]. Additionally, any disease-modifying treatment that will be developed will not remove the need for effective prevention of the disease [4], and with that the interest in accurate identification of people at risk.

External validation of a model can be difficult due to differences between datasets. Strengths of the study include the large sample size and use of a model with few variables to predict dementia risk. The baseline characteristics of the Rotterdam Study and the Lifelines cohort sample were largely similar, which is beneficial to validation of the model. We selected the predictors included in our analysis based on the results of the original Rotterdam Study. That model purposefully emphasized a limited set of readily obtainable variables in order to generate a basic version that could be applied by general practitioners outside specialized clinical care settings. An extended version of the Rotterdam model incorporates additional predictors, such as cognitive testing, brain MRI parameters, and genetic data, which were not available in the present study. As the majority of participants are of white European ancestry (98.9 %), the results are potentially less generalizable to other ancestries. While dementia is included as one of the potential outcomes in the Lifelines questionnaires, the Lifelines cohort study was set up as a biobank study, not as a dementia cohort which could influence registration of dementia cases. Additionally, the participants in this study are relatively young, with age ranging from 65–93 and a mean age of 69.7 years, thus follow-up time for many of the participants could be too short to identify dementia cases.

In our study, we do not address the implementation barriers and facilitators of dementia risk prediction models, although this is an issue of great importance. Significant efforts are currently underway to study dementia prevention and to work on implementation strategies [25,26]. While many dementia risk prediction models have been developed, their uptake in clinical practice remains limited. Recently, public attitudes towards dementia prevention risk models have been investigated [27]. The investigation included focus groups and an online survey to explore public perceptions of risk prediction. The focus groups revealed a reluctance to know one’s dementia risk, driven by fear, emotional burden, and the belief that prevention is not possible. However, some participants expressed motivation due to practical benefits and a desire to maximize their current quality of life. The survey found that 66.1 % of respondents wanted to know their 10-year dementia risk at that moment, increasing to 82.3 % if effective preventive medication were available. Most participants believed that a healthy lifestyle could reduce dementia risk. The study emphasized that the successful development and communication of dementia risk prediction tools must address emotional impacts and ensure that the information is personally actionable and supported by healthcare professionals. The authors also conclude that involving target users in the development of dementia risk prediction models is crucial for their effective clinical implementation [27].

The practical use of dementia risk models depends on many aspects. Importance should be given to their integration into routine consultations (such as annual check-ups, chronic disease management) without adding complexity or disrupting the use of electronic health records. Clinician acceptability requires tools that are easy to use, protocol-driven, and supported by appropriate training, considering time and workload pressures [28]. Ethical concerns and unclear follow-up pathways after identifying high-risk patients also limit adoption. Pilot studies in Dutch general practices show general support from both patients and clinicians but highlight ongoing organizational and infrastructural challenges that must be addressed for sustainable implementation [29]. The Rotterdam model, on which our analysis was based, deliberately emphasized a basic set of readily obtainable predictors to maximize feasibility in non-specialist settings. The following candidate predictors were initially selected by the Rotterdam model: age, sex, education level, systolic blood pressure, smoking, history of diabetes, history of stroke, presence of depressive symptoms, parental history of dementia, presence of subjective memory decline, and need for assistance with finances or medication. The discriminative accuracy measured with the C-statistic of the full basic model was 0.79 (95 % CI=0.76, 0.83). After shrinkage and predictor selection using the LASSO technique, these four predictors remained in the model: age, history of symptomatic stroke, presence of subjective memory decline, and need for assistance with finances or medication, resulting in a similar C-statistic remained similar (0.79; 95 % CI=0.76, 0.82). While this parsimony is a clear strength for accessibility and implementation, it also highlights an important limitation: a “true primary care ready tool” would need to incorporate a broader range of comorbidities and patient history beyond stroke, in order to better reflect the multimorbidity and clinical complexity commonly encountered in general practice. Additionally, social determinants of health and longitudinal patient data may further enhance predictive accuracy in real-world primary care populations. Finally, we wish to underscore that our work represents an external validation step of the Rotterdam model, rather than a definitive clinical screening tool. Future model development should focus on expanding predictor sets, integrating comorbidities, and ensuring performance across diverse patient groups before considering translation into routine clinical use. Considerations for the implementation of risk reduction trials should also consider acceptability which depends on clear and supportive communication that addresses fear, stigma, and the personal relevance of risk [29]. Resource requirements, including adequate digital infrastructure, staff time for counselling, and financial reimbursement, also remain common barriers.

Focusing on facilitators, strong leadership and organizational support help prioritize dementia risk assessment within clinical workflows. Adequate and ongoing training programs for clinicians increase confidence and competence in using these tools. The use of user-friendly, standardized protocols and digital tools that integrate with existing health IT systems enhances workflow efficiency and reduces disruptions. Furthermore, engaging patients through education and shared decision-making fosters trust and empowers individuals to act on their risk information. Positive feedback loops, where patients experience benefits from early risk awareness and preventive measures, can motivate both patients and clinicians to embrace these models.

In summary, the prediction model originally developed in the Rotterdam Study tended to overestimate dementia prevalence when applied to the Lifelines cohort, although it demonstrated acceptable discriminatory performance. However, the model's potential may be undervalued in this validation cohort due to the relatively low number of reported dementia cases. Because of this limited case number, we were unable to conduct additional exploratory analyses, as they would not have produced stable or interpretable estimates. As a sensitivity note, we acknowledge that such analyses would likely have been underpowered, and our findings should therefore be interpreted with this limitation in mind. Additional validation is needed to more accurately assess the model’s effectiveness and efforts are needed to further investigate barriers and facilitators for its implementation.

## Data availability

Data may be obtained from a third party and are not publicly available. Researchers can apply to use the Lifelines data used in this study. More information about how to request Lifelines data and the conditions of use can be found on their website

## Funding

The Netherlands Consortium of Dementia Cohorts (NCDC), is funded in the context of Deltaplan Dementie from ZonMW Memorabel and Alzheimer Nederland (project number 73305095005). The Lifelines initiative has been made possible by subsidy from the Dutch Ministry of Health, Welfare and Sport, the Dutch Ministry of Economic Affairs, the University Medical Center Groningen (UMCG), Groningen University and the Provinces in the North of the Netherlands (Drenthe, Friesland, Groningen).

## Ethics approval

This study was performed in line with the principles of the Declaration of Helsinki. The Lifelines study was approved by the ethics committee of the University Medical Center Groningen, document number METC UMCG METc 2007/152.

## Consent to publish

Informed consent was obtained from all individuals included in the study.

## CRediT authorship contribution statement

**Ingeborg Frentz:** Writing – original draft, Visualization, Validation, Methodology, Formal analysis, Data curation, Conceptualization. **Sofia Marcolini:** Writing – review & editing, Visualization, Project administration. **Silvan Licher:** Writing – review & editing, Validation, Methodology, Conceptualization. **Peter Paul De Deyn:** Writing – review & editing, Supervision, Conceptualization. **Mohammad Arfan Ikram:** Writing – review & editing, Supervision, Resources, Project administration, Methodology, Funding acquisition, Conceptualization.

## Declaration of competing interest

The authors declare that they have no known competing financial interests or personal relationships that could have appeared to influence the work reported in this paper.
